# One glucokinase, two tales of GLP-1 and insulin secretion

**DOI:** 10.1093/lifemeta/loag033

**Published:** 2026-10-12

**Authors:** Xu Wang, Tong-Jin Zhao

**Affiliations:** School of Life Science, Anhui Medical University, Hefei, Anhui 230032, China; Department of Endocrinology, Research Center for Translational Medicine, the Second Affiliated Hospital of Anhui Medical University, Hefei, Anhui 230032, China; State Key Laboratory of Metabolic Dysregulation & Prevention and Treatment of Esophageal Cancer, Innovation Center of Basic Research for Metabolic-Associated Fatty Liver Disease, Ministry of Education of China, Tianjian Laboratory of Advanced Biomedical Sciences, Academy of Medical Sciences, Zhengzhou University, Zhengzhou, Henan 450001, China; State Key Laboratory of Genetics and Development of Complex Phenotypes, Shanghai Key Laboratory of Metabolic Remodeling and Health, Institute of Metabolism and Integrative Biology, Department of Endocrinology and Metabolism, Zhongshan Hospital, Fudan University, Shanghai 200438, China

**Glucose sensing in intestinal L cells has long been attributed to GK-catalyzed ATP production. A recent study in *Vita* revises this model, showing that glucose-bound GK closes K_ATP_ and triggers GLP-1 secretion without an ATP rise, separating nutrient sensing from metabolic permission and revealing how the same sensor is read in two distinct modes.**Glucose is both a fundamental metabolic fuel and a major nutritional cue coordinating whole-body energy homeostasis. Following nutrient ingestion, glucagon-like peptide-1 (GLP-1) is released from enteroendocrine L cells to orchestrate postprandial metabolism through actions on pancreatic islets, the gastrointestinal tract, the nervous system, and other tissues [1–3]. For decades, glucose-triggered GLP-1 secretion in L cells has largely been viewed through the canonical framework of metabolic–electrical coupling. In this model, glucose enters the cell and is phosphorylated by glucokinase (GK), fueling glycolysis and mitochondrial oxidation. The resulting increase in the ATP/ADP ratio closes ATP-sensitive potassium (K_ATP_) channels, depolarizes the plasma membrane, and ultimately promotes Ca^2+^-dependent hormone exocytosis. This framework closely resembles the textbook model of glucose-stimulated insulin secretion in pancreatic β cells [4–7]. Classic electrophysiological studies established K_ATP_ channels as a crucial bridge between glucose metabolism and β-cell electrical activity [4, 5], embedding a seemingly intuitive assumption into the field that GK acts primarily as a metabolic enzyme and that glucose must be metabolized before it can become a secretory signal.

A recent study by Li *et al.* [8] in *Vita* fundamentally revises this assumption. The authors show that postprandial concentrations of glucose stimulate GLP-1 secretion from intestinal L cells without a detectable increase in cellular ATP. More strikingly, a nonmetabolizable glucose analog can still stimulate secretion provided that it forms a stable glucose-like complex with GK. By combining disease-associated GK mutants, biochemical interaction assays, Förster resonance energy transfer (FRET)-fluorescence lifetime imaging microscopy (FLIM), and electrophysiology, the study demonstrates that glucose-occupied GK directly engages the Kir6.2 subunit of K_ATP_ and suppresses channel activity. This distinguishes a requirement for basal ATP binding from a requirement for an acute rise in cellular ATP. Mutants that uncouple glucose binding from glucose catalysis further establish that ligand occupancy, rather than catalytic flux, is sufficient to support this secretory signal [8]. Thus, at least in L cells, glucose-triggered GLP-1 secretion can be experimentally separated from a rise in ATP and from glucose metabolism. Requiring high-glucose occupancy of GK may also prevent ATP generated from other nutrients from being sufficient, by itself, to drive robust GLP-1 release.

This separation raises a question that may be even more consequential than the newly defined L-cell pathway itself. If glucose-bound GK can regulate K_ATP_ without increasing ATP, why do pancreatic β cells retain the longer route of GK catalysis, glucose oxidation, and ATP production? The answer lies in the fundamentally different physiological tasks assigned to these two endocrine cell types. Intestinal L cells must rapidly detect that nutrients have arrived and are therefore optimized as nutrient sensors. Pancreatic β cells, in contrast, must determine whether the systemic metabolic state truly warrants a potent glucose-lowering response and therefore act as metabolic gatekeepers. At postprandial concentrations, glucose therefore serves distinct roles in the two cells: in intestinal L cells, it acts as a ligand, whereas in pancreatic β cells, it is metabolized as fuel.

The metabolic architecture of the two cell types is consistent with the division of labor. L cells can draw on pyruvate, lactate, fatty acids, and other substrates to support basal energy production, consistent with the stable intestinal adenylate levels observed between fasting and early refeeding and helping to explain why an acute increase in ATP is not obligatory for high-glucose-triggered GLP-1 release [8]. β cells, by contrast, are metabolically specialized to preserve a tight relationship between glucose oxidation and insulin secretion. Expression of alternative metabolic routes is selectively restricted: *SLC16A1* and *LDHA*, which encode monocarboxylate transporter 1 (MCT1) and lactate dehydrogenase A (LDHA), respectively, are among the best-characterized “disallowed genes” in β cells [9, 10]. Their repression prevents circulating lactate or pyruvate from bypassing glucose-dependent metabolic coupling and from inappropriately generating a metabolic signal for insulin secretion. This specialization converts ATP production from a generic consequence of nutrient oxidation into a stringent readout of systemic glucose availability.

These observations suggest a two-gate model of gut-islet glucose control (Fig. 1). Gate 1 resides in intestinal L cells. During the postprandial period, high luminal glucose enters through glucose transporter 2 (GLUT2), occupies GK, and promotes GK–Kir6.2 interaction, thereby closing K_ATP_ and triggering GLP-1 release. At lower glucose concentrations, electrogenic glucose-Na^+^ transport through sodium-glucose cotransporter 1 (SGLT1) contributes to basal GLP-1 secretion [11, 12]. This intestinal gate provides antici­pation: it reports that carbohydrate has reached the gut. Gate 2 resides in pancreatic β cells. Circulating GLP-1 engages the GLP-1 receptor (GLP-1R) and cyclic AMP (cAMP)-dependent pathways to potentiate secretory competence [1–3], but robust insulin secretion still requires elevated blood glucose, GK-catalyzed metabolism, mitochondrial ATP generation, K_ATP_ closure, and Ca^2+^ entry [4–7]. This β-cell gate provides authorization: it verifies that the systemic glucose state actually warrants insulin release. GLP-1 therefore amplifies the response, whereas ATP serves as a final metabolic permission signal. Because GLP-1 secretion can also be stimulated by nutrients other than glucose, retaining a requirement for glucose metabolism in β cells may help limit inappropriate insulin release and hypoglycemia when incretin signaling is elevated independently of glucose.

**Figure 1:**
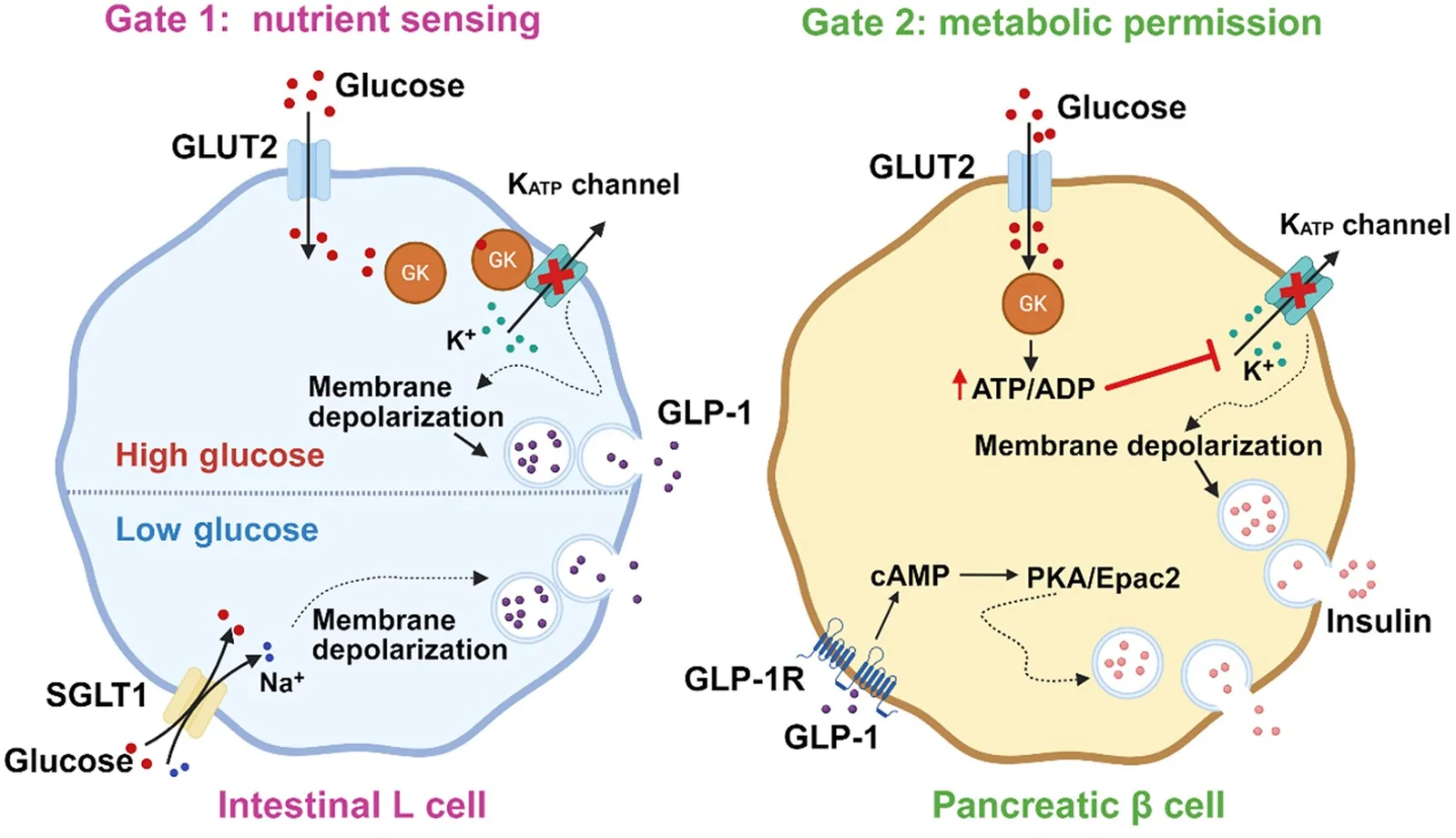
Distinct roles of GK in GLP-1 and insulin secretion. Postprandial high glucose enters intestinal L cells and directly occupies GK. Glucose-bound GK interacts with Kir6.2/K_ATP_, promoting channel closure, membrane depolarization, and GLP-1 secretion without requiring an increase in ATP. Circulating GLP-1 subsequently potentiates pancreatic β-cell secretory signaling through GLP-1R. However, insulin release remains metabolically gated: elevated blood glucose must be catalyzed by GK and oxidized to raise the ATP/ADP ratio, close K_ATP_, and trigger insulin exocytosis. The intestinal and pancreatic gates therefore provide sequential nutrient anticipation and metabolic authorization, respectively.

The study also encourages a broader reconsideration of how GK couples glucose to hormone secretion. In pancreatic β cells, GK catalysis generates metabolic flux and an ATP signal [6, 7]. In L cells, by contrast, glucose occupancy of GK alters protein interactions and directly controls downstream signaling [8]. Conceptually, this echoes previous work showing that the glycolytic metabolite fructose-1,6-bisphosphate communicates glucose availability through an aldolase-dependent signaling mechanism upstream of AMPK [13]. These findings raise the possibility that metabolic enzymes encode nutrient information not only through the products and fluxes they generate, but also through ligand-induced conformational states. GK and K_ATP_ are co-expressed in several additional endocrine and neuroendocrine cell populations, including pancreatic δ cells, enteroendocrine K cells, pituitary cells, and glucose-responsive neurons [14]. Determining whether ligand-like GK–K_ATP_ coupling operates in these settings will establish whether L cells represent an exception or the first clearly resolved example of a broader endocrine ligand-dependent GK–K_ATP_ coupling mechanism.

Separating GK catalysis from GK signaling also opens a different therapeutic space. Conventional glucokinase activators (GKAs) are designed to enhance glucose responsiveness through altered GK catalytic properties, and the dual-acting GKA dorzagliatin has demonstrated glucose-lowering efficacy in a phase 3 trial in type 2 diabetes [15]. However, the new mechanism suggests that increasing systemic glucose metabolism may not be necessary to exploit GK for incretin therapy. One possibility is a gut-restricted, pathway-biased GK modulator that preferentially stabilizes the glucose-bound signaling conformation of GK in intestinal L cells while minimizing sustained activation of hepatic or pancreatic glucose metabolism. A second, more mechanistically focused strategy would target the GK–Kir6.2 interface itself, using small molecules, allosteric stabilizers, or other approaches to favor the signaling complex that promotes endogenous GLP-1 secretion. Such approaches remain speculative, but they shift the therapeutic question from “how can GK catalysis be increased?” to “can the signaling output of GK be selectively biased?”

The lasting significance of the work by Li *et al.* may therefore extend beyond assigning a new molecular pathway to GLP-1 secretion. The study reveals a previously underappreciated hierarchy in glucose-dependent secretory control. Intestinal L cells read the presence of glucose, whereas pancreatic β cells verify its metabolic value. One anticipates nutrient arrival; the other authorizes insulin execution. Within this two-gate architecture, high glucose acts as a ligand in intestinal L cells but as a metabolic substrate in pancreatic β cells, and the output of GK is determined by the cellular context in which it operates. The emerging question is no longer simply how cells metabolize glucose, but how they decide when glucose should be interpreted as a signal and when it must first be consumed as a substrate.
